# Phase II Trial Evaluating Olaparib Maintenance in Patients with Metastatic Castration-Resistant Prostate Cancer Responsive or Stabilized on Docetaxel Treatment: SOGUG-IMANOL Study

**DOI:** 10.3390/cancers15215223

**Published:** 2023-10-31

**Authors:** María José Juan Fita, Urbano Anido Herranz, María José Mendez-Vidal, Regina Gironés-Sarrió, José Muñoz-Langa, Juan Sepúlveda-Sánchez, Begoña Mellado, Carlos Alvarez-Fernandez, Lucía Heras López, José Antonio López-Guerrero, Zaida García-Casado, Ana Calatrava, Miguel Ángel Climent

**Affiliations:** 1 Fundación Instituto Valenciano de Oncología, Carrer del Professor Beltrán Báguena, 8, 46009 Valencia, Spain; macliment@fivo.org; 2 Medical Oncology, Complejo Hospitalario Universitario de Santiago, 15706 Santiago de Compostela, Spain; urbano.anido.herranz@sergas.es; 3 Medical Oncology, Maimonides Institute for Biomedical Research of Córdoba (IMIBIC), Hospital Universitario Reina Sofía (HURS), 14004 Córdoba, Spain; mjosemv@yahoo.es; 4 Medical Oncology, Hospital Universitari i Politècnic la Fe, 46026 Valencia, Spain; girones_reg@gva.es; 5 Medical Oncology, Hospital Arnau de Vilanova, 46015 Valencia, Spain; munyoz_joslan@gva.es; 6 Medical Oncology, Hospital Universitario 12 de Octubre, Instituto de Investigación 12 de Octubre, 28041 Madrid, Spain; sepulvedasanchez@seom.org; 7 Medical Oncology, Hospital Clinic of Barcelona, 08036 Barcelona, Spain; bmellado@clinic.cat; 8 Translational Genomics and Targeted Therapies in Solid Tumors, August Pi i Sunyer Biomedical Research Institute (IDIBAPS), 08036 Barcelona, Spain; 9 Medical Oncology, Hospital Universitario Central de Asturias, Instituto de Investigación Sanitaria del Principado de Asturias, 33011 Oviedo, Spain; carlos.alvfer@gmail.com; 10 Servicio de Oncología Médica, Unidad de Sarcomas, Melanoma y Genitourinario, Institut Català d’Oncologia, 08908 L’Hospitalet de Llobregat, Spain; lheras@iconcologia.net; 11 Laboratory of Molecuar Biology, Fundación Instituto Valenciano de Oncología, 46009 Valencia, Spain or ja.lopez@ucv.es (J.A.L.-G.); zgarcia@fivo.org (Z.G.-C.); 12 Department of Pathology, Catholic University of València, 46001 Valencia, Spain; 13 Servicio Anatomía Patológica, Fundación Instituto Valenciano de Oncología, 46009 Valencia, Spain; acalatrava@fivo.org

**Keywords:** castration-resistant prostate cancer, docetaxel, maintenance therapy, olaparib

## Abstract

**Simple Summary:**

In this study, based on the results of chemotherapy or poly(ADP-ribose) polymerase (PARP) inhibitor (PARPi) maintenance in other tumors, we explore whether olaparib, a PARP inhibitor, could be useful in terms of prolonging radiographic progression of the disease in patients with metastatic castration-resistant prostate cancer with specific mutations whose illness had not progressed under treatment with docetaxel—A standard chemotherapy for these patients. In the 14 patients included in this study harboring mutations in homologous recombination genes, olaparib maintenance was an effective option, stabilizing the metastasis and extending the radiographic and clinical progression of the disease with tolerable and manageable adverse events. Overall, the results suggest this alternative could be useful for selected patients.

**Abstract:**

The SOGUG-IMANOL trial was a phase 2, uncontrolled, Spanish multicenter study to assess the effect of maintenance treatment with olaparib on radiographic progression-free survival (PFS) in patients with metastatic castration-resistant prostate cancer (mCRPC) who achieved partial or complete response or disease stabilization on docetaxel treatment and had a documented germline/somatic mutation in any of the homologous recombination repair (HRR) genes. Patients received olaparib 300 mg orally twice daily. From the screened population (*n* = 134), 26 (19.4%) somatic mutations were found, and 14 patients were included in the study. The median radiographic PFS was 11.1 (95%CI, 5.7 to 16.5) months. The median PSA-PFS was 3.5 (95%CI, 1.0 to 6.0) months, and the median clinical PFS was 14.7 (95%CI, 1.8 to 27.5 months). Clinical benefit was observed in 12 patients (85.7%, 95%CI 67.4% to 100%), including two patients with partial response and 10 with stable disease. Six patients reported grade 3–5 adverse events: asthenia (*n* = 3), anemia (*n* = 2) and neutropenia (*n* = 1). In this setting, olaparib has been shown to be an efficacious maintenance treatment in terms of radiographic PFS and clinical benefit, becoming a therapeutic option for some patients harboring an HRR gene mutation and in scenarios where further investigation is needed.

## 1. Introduction

Prostate cancer (PC) is, after lung cancer, the second most common cause of cancer in men (14.1% of all cases in 2020) and the fifth leading cause of death (6.8% of all cancer deaths in 2020) [1].

Treatment of PC follows a stage-matched strategy [2,3]. Patients with localized disease may be treated definitely with radiotherapy or surgery. Men who are diagnosed with or progress to metastatic PC, an incurable entity, are usually treated with androgen deprivation therapy (ADT) combined with new hormonal agents such as abiraterone, enzalutamide or apalutamide, or, based on the results of the LATITUDE [4,5], ARCHES [6], ENZAMET [7], TITAN [8], PEACE1 [9], and ARASENS [10] trials, with double and triple therapies, which have shown a significant clinical benefit in prolonging disease control. However, PC progresses invariably to castration-resistant PC (mCRPC), and additional systemic therapies to delay disease progression in these patients are required.

The treatment landscape of mCRPC has evolved in recent years, and currently, several systemic therapies, such as docetaxel, cabazitaxel, abiraterone, enzalutamide, radium223, and olaparib, have been developed for this scenario, having shown benefits in terms of overall survival and having received regulatory approval [2,3]. Nevertheless, the optimal sequence of these treatments is unknown [2,11], and the decision is based on disease extension, comorbidities, previous treatments, patients’ preferences, physician experience, and drug availability [2].

In this setting, we usually offer a new treatment line to a patient once the disease has progressed. However, based on the results of chemotherapy, immunotherapy or poly(ADP-ribose) polymerase (PARP) inhibitors (PARPis) maintenance in other tumors such as non-small-cell lung cancer [12,13], pancreatic cancer [14], bladder cancer [15,16], and ovarian cancer [17], this strategy could be an option. In patients with mCRPC, trials with sunitinib, orteronel, and tasquinimod have explored this option with mixed results [18,19,20].

More recently, PARPis have been added to the list of therapeutic alternatives based on the efficacy of these drugs in patients with mCRPC harboring somatic or germline alterations of the homologous recombination repair (HRR) genes such as *BRCA1*, *BRCA2*, *ATM*, and *CHEK2* [21]. Thus, based on the results of the PROfound trial with olaparib [22], clinical practice guidelines include this agent as an option for the management of patients with mCRPC and HRR gene alterations who have progressed to a new hormonal therapy [2,23,24], and due to the PROPEL [25], MAGNITUDE [26], and TALAPRO-2 [27] trial results, PARPis in combination with abiraterone or enzalutamide have shown to be efficacious in the first-line treatment of patients with mCRPC.

Regarding maintenance treatment, olaparib has shown to be efficacious in patients with pancreatic [14] and ovarian cancer [17], and docetaxel has been and remains an effective option inside the sequence for this mCRPC patients. As such, the SOGUG-IMANOL trial was designed to assess the effect of maintenance treatment with olaparib on radiographic PFS in patients with mCRPC who had received at least six cycles of docetaxel and achieved partial or complete response or disease stabilization and had a documented germline/somatic mutation in any of the HRR genes. Secondary objectives included the impact of olaparib on PSA-PFS, clinical PFS, radiological response rate, and PSA response rate; tolerability and safety were also assessed.

## 2. Materials and Methods

### 2.1. Study Design

This was a phase 2, uncontrolled Spanish multicenter study. The study was conducted following the principles included in the Declaration of Helsinki and the Good Clinical Practice guidelines. It was reviewed and approved by the ethics committee of the Hospital Universitario 12 de Octubre (Madrid, Spain; reference Nr. CEIm 17/390). Before any study procedure, all patients gave their written informed consent. The study was registered at ClinicalTrials.gov (NCT03434158).

### 2.2. Patients

Eligible patients were males aged 18 years or older with histologically confirmed prostate adenocarcinoma who had metastatic disease (documented by positive bone scan or metastatic lesion on computed tomography (CT) or magnetic resonance imaging (MRI)), with no prior exposure to platinum, cyclophosphamide, mitoxantrone or PARP inhibitors, and who did not progress after at least six cycles and a maximum of ten cycles of chemotherapy with docetaxel-based on the Prostate Cancer Working Group 3 (PCWG3) criteria. In addition, patients had adequate organ and bone marrow function and European Cooperative Oncology Groups (ECOGs) performance status of 0–1, and had documented germline/somatic mutation in any of the HRR genes, including *ATM*, *BARD1*, *BRCA1*, *BRCA2*, *BRIP1*, *CDK12*, *CHEK1*, *CHEK2*, *FANCL*, *PALB2*, *PPP2R2A*, *RAD51B*, *RAD51C*, *RAD51D*, and *RAD54L*, that were predicted to be deleterious or suspected deleterious, excepting genetic variants of unknown significance. Patients who had received treatment for mCRPC before docetaxel (abiraterone, enzalutamide, radium 223, etc.) or who had received prior docetaxel in a hormone-sensitive setting were also allowed for inclusion. The full list of exclusion criteria is presented in the Supplementary Table S1.

### 2.3. Intervention

After confirming eligibility criteria, patients received a standard dose of olaparib tablets (i.e., 300 mg twice daily). Treatment was initiated within seven days of inclusion in the study and not later than 56 days after the last dose of docetaxel. The dose of olaparib could be reduced up to 200 mg twice daily depending on the toxicity or to 150 or 100 mg twice daily if the patient unavoidably required treatment with a moderate or strong inhibitor of CYP3A4, respectively. Treatment continued until progression or unacceptable toxicity. Other anti-cancer therapies, including chemotherapy, radiotherapy, immunotherapy, or treatment with novel agents, were not allowed except for palliative radiotherapy for the treatment of pain at the site of the bone metastases that were present at baseline, providing the investigator did not consider that pain was indicative of clinical progression during the study period.

### 2.4. Study Assessments

All screening assessments to qualify the patient for entering into the study had to be performed within 28 days before Day 1 Cycle 1 of olaparib, except for tumor assessment, which could be conducted within 56 days prior to Day 1 Cycle 1 of olaparib. All patients had to undergo a next-generation sequencing panel analysis of the HRR genes centrally after the first infusion of docetaxel and before enrollment into the study. Sequencing was carried out with the Homologous Recombination Solution (HRS; Sophia Genetics, Rolle, Switzerland) capture kit in the Illumina MiSeq^®^ and NextSeq550^®^ sequencers. The study includes the analysis of the entire coding region and adjacent intronic regions (±25 pb) of 16 genes involved in HRR: *ATM*, *BARD1*, *BRCA1*, *BRCA2*, *BRIP1*, *CDK12*, *CHEK1*, *CHEK2*, *FANCL*, *PALB2*, *PPP2R2A*, *RAD51B*, *RAD51C*, *RAD51D*, *RAD54L*, and *TP53*. Bioinformatic analysis was undertaken with the analysis software and algorithms developed by Sophia Genetics (Sophia DDM^TM^), as well as with the support of other bioinformatic tools such as the Integrative Genome Viewer (IGV; www.broadinstitute.org, Cambridge, MA, USA). The analysis did not include large rearrangements. The sensitivity limit was set at 5% for point variants and 10% for insertion or deletion variants. The minimum coverage to consider a region properly covered was 600 readings (600X). In cases where a tumor study was not possible, a germline study was carried out.

Disease progression was evaluated every 12 weeks during the treatment period by means of CT or MRI of the chest, abdomen, and pelvis and a bone scan; if progressive disease was observed on a bone scan at any timepoint without progression on CT/MRI via the RECIST 1.1 criteria, a confirmatory bone scan performed 6 weeks later was required. Prostate-specific antigen (PSA) was evaluated at the screening, every cycle during treatment, and at the end of treatment. Safety and tolerability assessments, including physical examination, vital signs, hematology and biochemistry, and recording of adverse events were performed at the screening, every cycle during treatment, end of treatments, and a safety follow-up visit that took place 30 days after the end of treatment.

### 2.5. Endpoints

The primary endpoint was radiographic PFS, defined as the time from the start of olaparib treatment to radiographic progression or death by any cause. Radiographic progression was defined according to RECIST 1.1 and PCWG3.

Secondary efficacy endpoints included PSA-PFS, defined as the time from the start of treatment with olaparib to the date of first PSA progression, as evaluated with the PWCG3 criteria or death by any cause; clinical PFS, defined as the time from the start of treatment with olaparib to the date of first clinical progression or death by any cause; clinical progression, defined as a significant pain increase as judged by the investigator or a clinical deterioration that, in the investigator’s judgment, required initiating another line of treatment; radiologic response rate, defined as the proportion of patients fulfilling RECIST 1.1 criteria and evaluated among patients who had measurable disease at baseline; and PSA response rate, defined as a reduction of at least 50% in the concentration of PSA. Safety was evaluated through the reporting of adverse events according to the Common Terminology Criteria Adverse Events, version 4.

### 2.6. Statistical Analysis

Sample size estimation was based on the results of previous studies that had shown that the median rPFS in patients receiving docetaxel as a first-line treatment was about 2.8 months after the end of treatment with docetaxel [19]. Bearing in mind the results of olaparib maintenance in patients with ovarian cancer (SOLO1 study) [17], and the study reported in previously treated mCRPC with a PFS of 9.8 months after treatment with olaparib, it was decided to accept the treatment efficacy if the median radiographic PFS with olaparib maintenance was at least 6 months. An overall sample size of 16 patients was estimated to provide 80% power at a 0.05 significance alpha level to accept the efficacy of olaparib monotherapy maintenance after completion of the standard systemic treatment.

Time-to-event data were analyzed via the Kaplan–Meier method, which allows the estimation of the medians and corresponding 95% confidence intervals (CI). Binary outcomes were presented using the absolute and relative frequency with the corresponding 95%CI.

All analyses were performed using IBM SPSS version 26.

## 3. Results

### 3.1. Patient Disposition and Characteristics and Treatment with Olaparib

From February 2018 to November 2020, 134 patients were screened looking for germline/somatic HRR gene alterations, and 14 patients were included in the study; among the 120 who were excluded, 103 were excluded due to no pathogenic mutations, 7 due to disease progression after or during docetaxel, 3 due to death, and 7 for other reasons (i.e., investigator’s decision, subject withdrawn, or insufficient material). All 14 patients were included in the efficacy and safety analyses.

The median (interquartile range) age was 73.0 (72.0–75.0); 8 (43%) of the patients showed a Gleason score equal to or greater than 8, and 10 (71%) had an ECOG of 0. Four (28.6%) had visceral metastases. From the screened population, 26 (19.4%) HRR gene mutations were found; 12 of these patients had screening failures. The most common HRR gene defect in the 14 patients included in the study was *ATM* (*n* = 5, 36%), followed by *CHEK2* (*n* = 2, 14%) and *BRCA2* (*n* = 2, 14%). The best response to docetaxel treatment was stable disease in all patients. The detailed demographic and clinical characteristics of the patients included in the analyses are shown in Table 1.

### 3.2. Efficacy

**Figure 1:**
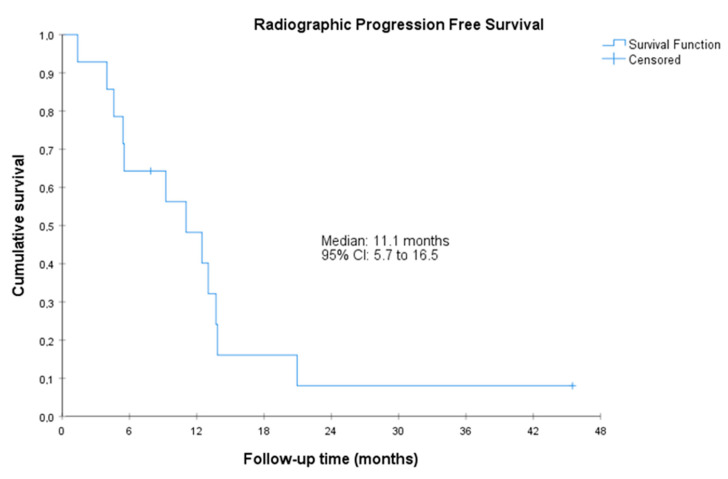
Radiographic progression-free survival. CI, confidence interval. Radiographic PFS was defined as the time from the start of treatment to radiographic progression or death by any cause. Radiographic progression was defined according to RECIST 1.1 and PCWG3.

**Figure 2:**
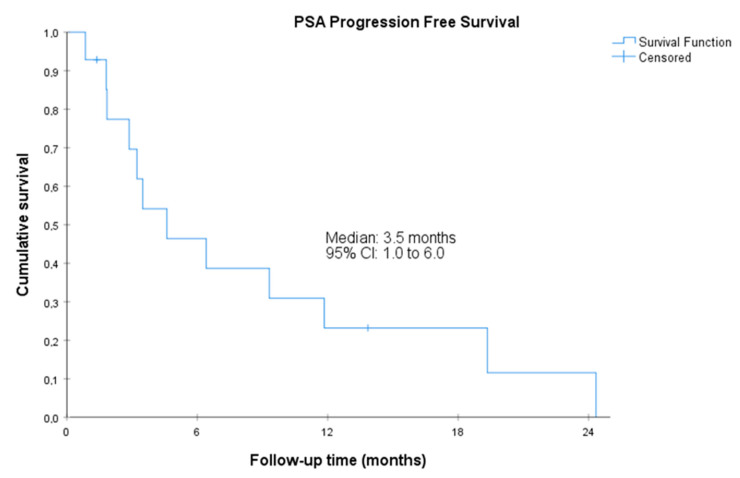
Prostate-specific antigen progression-free survival. CI, confidence interval. Prostate-specific antigen PFS was defined as the time from the start of treatment with olaparib to the date of first PSA progression as evaluated with the PWCG3 criteria or death by any cause.

**Figure 3:**
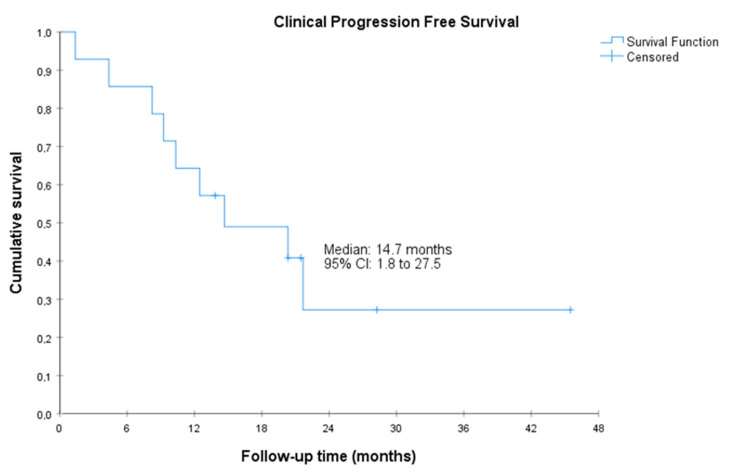
Clinical progression-free survival. CI, confidence interval. Clinical PFS was defined as the time from the start of treatment with olaparib to the date of first clinical progression or death by any cause (clinical progression was defined as a significant pain increase as judged by the investigator or a clinical deterioration that, in the investigator’s judgment, required initiating another line of treatment).

The median radiographic PFS was 11.1 (95%CI, 5.7 to 16.5) months. The median PSA-PFS was 3.5 (95%CI, 1.0 to 6.0) months, and the median clinical PFS was 14.7 (95%CI, 1.8 to 27.5 months) (Figure 1, Figure 2 and Figure 3). Clinical benefit was observed in 12 patients (85.7%, 95%CI 67.4% to 100%), including 2 patients with partial response (i.e., an objective response rate of 14.3%, 95%CI, 0.0 to 32.6) and 10 patients (71.4%) with stable disease. Two (14.3%, 95%CI 0.0 to 32.6) achieved PSA response. Individual patient data for efficacy outcomes and some demographic and clinical characteristics are shown in Supplementary Table S2.

### 3.3. Safety

Overall, 13 (92.9%) of the patients reported adverse events. The most frequent adverse events were asthenia (*n* = 10, 71.4%), anemia (*n* = 9, 64.3%), and nausea (*n* = 8, 57.1%) (Table 2). Six patients reported grade 3–5 adverse events: three patients reported asthenia, two patients reported anemia, and one patient had neutropenia. Two patients reported serious adverse events: one case of grade 3 urinary tract infection and one case of grade 4 bacteremia; both cases were categorized as serious because they required hospitalization, although they were not considered related to the study medication, and both patients recovered.

The median (interquartile range (IQR)) exposure to olaparib was 32.2 (19.0–64.3) months. There were ten dose reductions in eight patients (two patients required two dose reductions): in nine cases because of non-hematologic toxicity (mainly grade 2–3 asthenia [*n* = 4]) and one case because of a grade 2 platelet count decrease. There were ten dose interruptions in five patients: in six cases because of non-hematological toxicity (mainly grade 2–3 asthenia [*n* = 5]), in three cases because of hematological toxicity (grade 2 neutropenia (*n* = 1) and grade 2 platelet count decreased (*n* = 2)), and one dose interruption was considered unrelated to the study drug. The median (IQR) dose intensity of olaparib was 560.0 (448.5–600.0) mg/day. Individual patient data for key safety outcomes and some demographic and clinical characteristics are shown in Supplementary Table S3.

## 4. Discussion

Our results indicate that maintenance treatment with olaparib in patients with mCRPC and HRR gene defects who have achieved stabilization with docetaxel prolongs radiographic PFS. Benefits were also observed in terms of clinical PFS with an acceptable toxicity profile.

In patients with mCRPC, the time to progression after docetaxel treatment is short. In the TROPIC trial, the baseline data indicate that the median time to progression after the last dose of docetaxel was less than 1 month; moreover, almost 30% of the patients had progressed during treatment with docetaxel and over 40% within 3 months of the last dose of docetaxel [28]. Another maintenance trial evaluating orteronel (see below) showed that patients who were at least stabilized under docetaxel treatment and were randomized to placebo had a median time to progression of 2.9 months (with progression defined as time to death or the combination of at least two of radiographic, clinical or PSA progression) [19]. Hence, our results suggest that maintenance treatment with olaparib could significantly extend the time to progression in patients harboring a somatic or germline HRR gene mutation. The median time to clinical progression (evaluated from the start of olaparib maintenance treatment after docetaxel treatment) in our study was 14.7 months, although the imprecision was large (95%CI 1.8 to 27.5). Our results have been observed in a sample that seems representative of patients with mCRPC and HRR gene defects in our setting; thus, the overall frequency of HRR gene defects in our study was 19.4%, which is consistent with the 16% previously reported in the PROREPAIR-B study, a multicenter study also conducted in Spain [29].

Although a maintenance treatment strategy in this setting has been scarcely investigated, our efficacy results with olaparib are also favorable compared to all other treatment strategies tested. In a phase 2 study, sunitinib maintenance therapy failed to demonstrate any benefit in PFS in patients with mCRPC who responded or had stable disease after docetaxel treatment, showing a median PFS of 4.4 months and a median PSA-PFS of 2.8 months [18], while the median times to clinical progression and to PSA progression in our study were longer (14.7 and 3.3 months, respectively). In this setting, orteronel, an oral inhibitor of androgen biosynthesis whose development was discontinued, showed a significant improvement in event-free survival as defined above, with a median time to progression of 8.5 months [19]; the investigators of this trial also reported a median time to radiographic progression of 8.5 months, which is shorter than that achieved with olaparib in our study (11.1 months). A third trial with tasquinimod, an agent with immunomodulatory and antiangiogenic properties, also showed a significantly increased radiographic PFS compared to placebo in this setting, but the median time to progression (31.7 weeks [approximately 7.5 months]) was also shorter than with olaparib in our trial. Finally, in a recently reported placebo-controlled trial in patients with mCRPC, darolutamide, an androgen receptor inhibitor indicated for the treatment of metastatic hormone-sensitive prostate cancer in combination with docetaxel and androgen deprivation therapy, has shown significant benefit (but of doubtful clinical relevance) in the post-docetaxel maintenance treatment setting, with a median time to radiographic progression of 5.5 months, a median time to PSA progression of 2.7 months and a median time to symptomatic/clinical progression of 5.4 months [30]; all these outcomes are poorer than those achieved with olaparib in our study.

The toxicity profile observed in our study is fully consistent with that reported with olaparib in the PROfound trial [22], with asthenia, anemia, and nausea being the most frequent treatment-emergent adverse events. Grade 3–4 adverse events were reported in six (43%) of the patients, a finding which is also consistent with the results of the PROfound trial.

During the development of this study, further information on the use of olaparib for patients with mCRPC has been published, and some studies are currently ongoing. De Bono et al. [22] and Hussain et al. [31], in the PROfound trial, reported that olaparib in patients with mCRPC with alterations in *BRCA1*, *BRCA2*, or *ATM* genes who had disease progression while receiving enzalutamide and abiraterone (almost two-thirds of the patients had received a taxane) had a median time to imaging-based progression of 7.4 months with olaparib compared to 3.6 months with the physician’s choice of enzalutamide and abiraterone, with a significant benefit in terms of overall survival. This trial was the basis for the approved indication in Europe of olaparib monotherapy for the treatment of adult patients with mCRPC and BRCA1/2 mutations (germline and/or somatic) who have progressed following prior therapy that included a new hormonal agent [32]. Another trial using olaparib monotherapy (NCT04038502) is investigating the use of olaparib or carboplatin as first-line therapies with crossover to the alternate or second-line drug after first progression for patients with mCRPC harboring *BARD1*-, *BRCA1*-, *BRCA2*-, *BRIP1*-, *CHEK1*-, *FANCL*-, *PALB2*-, *RAD51B*-, *RAD51C*-, *RAD51D*-, or *RAD54L*-inactivating mutations [33]. Moreover, Clarke et al. [25] reported that the combination of olaparib with abiraterone significantly prolonged imaging-based PFS compared with abiraterone alone as a first-line treatment for patients with mCRPC enrolled irrespective of HRR mutation status. Currently, in Europe, olaparib is also indicated in combination with abiraterone and prednisone or prednisolone for the treatment of adult patients with mCRPC in whom chemotherapy is not clinically indicated [32]. Overall, we believed that maintenance treatment with olaparib in patients with mCRPC who have at least stabilized after docetaxel treatment may be an option for patients harboring HRR gene mutations, such as those who are unfit for cabazitaxel treatment, although the precise role of olaparib monotherapy or in combination for the management of mCRPC other than that approved by the EMA and included in the clinical practice guidelines should be further evaluated.

In addition to the small sample size and uncontrolled design, our study has the limitation of using a surrogate endpoint as a primary efficacy outcome: radiographic PFS. However, it is important to note that radiographic PFS has been shown to be clinically meaningful and correlated with overall survival [34,35].

## 5. Conclusions

In this phase II trial of patients with mCRPC with HRR mutations who had achieved radiological response or stabilization and had not progressed after docetaxel, olaparib was shown to be an effective maintenance treatment in terms of radiographic PFS and clinical benefit, becoming a therapeutic option for some patients and in scenarios where further investigation is needed.

## Figures and Tables

**Table 1:** Demographic and clinical characteristics.

Characteristic	N = 14
Age, median (IQR)	73.0 (72.0–75.0)
Time from the initial diagnosis (months), median (IQR)	61.4 (34.21–24.4)
Metastatic disease at initial diagnosis, *n* (%)	2 (14.3)
Time from diagnosis of metastatic disease (months), median (IQR)	24.6 (12.9–37.0)
Location of metastases, *n* (%)	
Bone	11 (78.6)
Lymph nodes	5 (35.7)
Liver	4 (28.6)
Prostate	1 (7.1)
Lung	1 (7.1)
Gleason score ≥ 8, *n* (%)	6 (42.8)
ECOG, *n* (%)	
0	10 (71.4)
1	4 (28.6)
Patients with HRR gene defects, *n* (%)	
*ATM*	5 (35.7)
*CHEK2*	2 (14.3)
*BRCA2*	2 (14.3)
*BRCA1*, *BRCA2*, *BRIP1*, *FANCL*, *PALB2*, *RAD51C*, *CDK12*, *TUBB3*	1 (7.1)
*CDK12*	1 (7.1)
*BRCA1*, *CDK12*	1 (7.1)
*BRCA1*, *BRCA2*	1 (7.1)
*BRCA1*	1 (7.1)
Previous hormonal agents, *n* (%)	
Bicalutamide	9 (64.3)
Triptorelin	7 (50.0)
Abiraterone	6 (42.9)
Enzalutamide	5 (35.7)
Goserelin	4 (28.6)
Other	5 (35.7)
Previous taxane use, *n* (%)	
Docetaxel	13 (92.9)
Docetaxel + Carboplatin	1 (7.1)
Duration of previous chemotherapy (months), median (IQR)	4.7 (3.5–5.2)
Best response under taxane, *n* (%)	
Stable disease	14 (100%)

ECOG, European Cooperative Oncology Groups; HHR, homologous recombination repair; IQR, interquartile range.

**Table 2:** Most frequent adverse events with maintenance olaparib.

	Grade							
	1		2		3–4 ^a^		Total	
	N	%	N	%	N	%	N	%
Asthenia	2	14.3	4	28.6	3	21.4	9	64.3
Anemia	4	28.6	3	21.4	2	14.3	9	64.3
Nausea	4	28.6	4	28.6	0	0.0	8	57.1
Neutropenia	2	14.3	2	14.3	1	7.1	5	35.7
Decreased appetite	3	21.4	2	14.3	0	0.0	5	35.7
Diarrhea	1	7.1	3	21.4	0	0.0	4	28.6
Edema, peripheral	3	21.4	1	7.1	0	0.0	4	28.6
Mucosal inflammation	1	7.1	2	14.3	0	0.0	3	21.4
Leukopenia	2	14.3	1	7.1	0	0.0	3	21.4
Vomiting	1	7.1	1	7.1	0	0.0	2	14.3
Abdominal pain, upper	2	14.3	0	0.0	0	0.0	2	14.3
Abdominal discomfort	2	14.3	0	0.0	0	0.0	2	14.3
Dyspnea	2	14.3	0	0.0	0	0.0	2	14.3
Cognitive disorder	2	14.3	0	0.0	0	0.0	2	14.3

Adverse events that were reported by at least two patients. ^a^ There were no cases of grade 5 adverse events.

## Data Availability

The data that support the findings of this study are available from the corresponding author upon reasonable request.

## Supplementary Materials

The following supporting information can be downloaded at: https://www.mdpi.com/article/10.3390/cancers15215223/s1, Table S1. Exclusion criteria; Table S2. Individual patient data for efficacy outcomes; Table S3. Individual patient data for key safety outcomes.
