# Supplementary material for: Phase II Trial Evaluating Olaparib Maintenance in Patients with Metastatic Castration-Resistant Prostate Cancer Responsive or Stabilized on Docetaxel Treatment: SOGUG-IMANOL Study
Source: Cancers (Basel). 2023 Oct 31;15(21):5223. doi: 10.3390/cancers15215223 (PMC10649956; doi:10.3390/cancers15215223)
Supplement: Supplementary file 1 [file cancers-15-05223-s001.zip › cancers-2665901-supplementary.pdf]

Supplementary Materials: Phase II Trial Evaluating Olaparib Maintenance in Patients with Metastatic Castration-resistant Prostate Cancer Responsive or Stabilized on Docetaxel Treatment: SOGUG-IMANOL Study

Table S1. Exclusion criteria

1. Involvement in the planning and/or conduct of the study (applies to AstraZeneca or sponsor staff and/or staff at the study site).
2. Previous inclusion in the present study.
3. Participation in another clinical study with an investigational product during the last month.
4. Any previous treatment with PARP inhibitor, including olaparib.
5. Patients who do not have deleterious or suspected deleterious Homologous Recombination Repair genes mutations and only have Homologous Recombination Repair genes mutations that are considered to be non-detrimental (e.g., "Variants of uncertain clinical significance" or "Variant of unknown significance" or "Variant, favour polymorphism" or "benign polymorphism" etc.).
6. Other malignancy within the last 5 years except: adequately treated non-melanoma skin cancer, curatively treated in situ cancer of the cervix, ductal carcinoma in situ (DCIS), Stage 1, grade 1 endometrial carcinoma, or other solid tumours including lymphomas (without bone marrow involvement) curatively treated with no evidence of disease for ≥5 years.
7. Resting ECG with QTc > 470 msec on 2 or more time points within a 24 hour period or family history of long QT syndrome.
8. Patients receiving any systemic chemotherapy or radiotherapy (except for palliative reasons) within 3 weeks prior to study treatment.
9. Concomitant use of known strong CYP3A inhibitors (eg. itraconazole, telithromycin, clarithromycin, protease inhibitors boosted with ritonavir or cobicistat, indinavir, saquinavir, nelfinavir, boceprevir, telaprevir) or moderate CYP3A inhibitors (eg. ciprofloxacin, erythromycin, diltiazem, fluconazole, verapamil). The required washout period prior to starting olaparib is 2 weeks.
10. Concomitant use of known strong (eg. phenobarbital, enzalutamide, phenytoin, rifampicin, rifabutin, rifapentine, carbamazepine, nevirapine and St John's Wort) or moderate CYP3A inducers (eg. bosentan, efavirenz, modafinil). The required washout period prior to starting olaparib is 5 weeks for enzalutamide or phenobarbital and 3 weeks for other agents.
11. Persistent toxicities (>Common Terminology Criteria for Adverse Event (CTCAE) grade 2) caused by previous cancer therapy, excluding alopecia and nail toxicity.
12. Patients with myelodysplastic syndrome/acute myeloid leukaemia or with features suggestive of MDS/AML.
13. Patients with symptomatic uncontrolled brain metastases. A scan to confirm the absence of brain metastases is not required. The patient can receive a stable dose of corticosteroids before and during the study as long as these were started at least 4 weeks prior to treatment. Patients with spinal cord compression unless considered to have received definitive treatment for this and evidence of clinically stable disease for 28 days.
14. Major surgery within 4 weeks of starting study treatment and patients must have recovered from any effects of any major surgery.
15. Patients considered a poor medical risk due to a serious, uncontrolled medical disorder, non-malignant systemic disease or active, uncontrolled infection. Examples include, but are not limited to, uncontrolled ventricular arrhythmia, recent (within 3 months) myocardial infarction, uncontrolled major seizure disorder, unstable spinal cord compression, superior vena cava syndrome, extensive interstitial bilateral lung disease on High Resolution Computed Tomography (HRCT) scan or any psychiatric disorder that prohibits obtaining informed consent.
16. Patients unable to swallow orally administered medication and patients with gastrointestinal disorders likely to interfere with absorption of the study medication.
17. Immunocompromised patients, e.g., patients who are known to be serologically positive for human immunodeficiency virus (HIV).
18. Patients with a known hypersensitivity to olaparib or any of the excipients of the product.
19. Patients with known active hepatitis (i.e. Hepatitis B or C) due to risk of transmitting the infection through blood or other body fluids.
20. Previous allogenic bone marrow transplant or double umbilical cord blood transplantation (dUCBT).
21. Whole blood transfusions in the last 120 days prior to entry to the study (packed red blood cells and platelet transfusions are acceptable).

Table S2. Individual patient data for efficacy outcomes.

Patient ID	Age	Time from diagnosis of metastatic disease (months)	Visceral metastases (yes/no)	Gleason score	ECOG. n (%)	HRR gene defects	Response	Radiographic PFS (months)	PSA-PFS (months)	Clinical-PFS (months)
1	83.0	74.7	Liver. Lung	7	1	ATM	Stable Disease	45.5	24.3	45.5
2	72.0	12.9	-	8	1	Fanconi genes, CDK12,T UBB3	Stable Disease	13.0	2.9	14.7
3	54.0	5.3	Liver	9	0	BRCA2	Stable Disease	5.4	1.8	20.3
4	80.0	32.7	-	6	0	ATM	Stable Disease	7.9	3.5	8.2
5	75.0	11.0	-	8	0	BRCA1, CDK12	Not Evaluable	1.4	1.4	1.4
6	72.0	18.8	-	6	0	CHEK2	Partial Response	11.1	13.8	13.8
7	75.0	35.3	Liver	8	0	BRCA1	Stable Disease	4.6	1.8	4.4
8	73.0	23.6	-	8	1	ATM	Stable Disease	13.8	11.8	28.3
9	72.0	41.8	-	7	0	CHEK2	Stable Disease	5.5	0.9	21.7
10	58.0	37.0	-	9	0	BRCA2	Stable Disease	21.0	19.3	21.5
11	75.0	19.9	-	7	0	ATM	Stable Disease	12.5	6.4	12.5
12	82.0	8.3	-	7	0	BRCA1, BRCA2	Progression Disease	4.0	3.2	10.3
13	55.0	53.1	-	7	0	ATM	Stable Disease	9.2	4.6	9.2
14	73.0	25.6	Liver	8	1	CDK12	Partial Response	13.7	9.3	20.3

Table S3. Individual patient data for key safety outcomes.

Patient ID	Age (years)	ECOG	Exposure to Olaparib (weeks)	Dose reduction (Yes/No)	Dose interruption (Yes/No)	Adverse events (list)
1	83.0	1	125.71	Yes	Yes	Grade 3: Asthenia.<br>Grade 2: Diarrhoea, Mucosal inflammation, Nausea.<br>Grade 1: Abdominal discomfort, Abdominal pain upper, Anaemia, Cognitive disorder, Decreased appetite, Dyspnoea, Nasopharyngitis, Oedema peripheral, Orthopnoea, Vertigo.
2	72.0	1	12.29	Yes	No	Grade 2: General physical health deterioration. Neutropenia, Oedema peripheral;<br>Grade 1: Asthenia, Diarrhoea.
3	54.0	0	28.43	No	No	Grade 2: Diarrhoea;<br>Grade 1: Nausea, Rectal tenesmus.
4	80.0	0	23.14	Yes	Yes	Grade 3: Asthenia;<br>Grade 2: Nausea;<br>Grade 1: Abdominal discomfort, Abdominal pain upper, Anaemia, Blood creatinine decreased, Cognitive disorder, Decreased apetite, Disorientation, Headache, Leukopenia, Neutropenia.

5	75,0	0	3,86	No	No	None
6	72.0	0	56.00	Yes	No	Grade 2: Anaemia, Blood creatinine increased;<br>Grade 1: Dysgeusia.
7	75.0	0	19.00	Yes	Yes	Grade 3: Anaemia;<br>Grade 2: Urinary tract infection; Asthenia;<br>Grade 1 Haematuria, Nausea.
8	73.0	1	64.29	No	No	Grade 4: Neutropenia;<br>Grade 2: Alopecia, Asthenia, Decreased appetite, Diarrhoea, Nausea, Pyrexia;<br>Grade 1: Anaemia, Hypomagnesaemia, Oedema peripheral, Vomiting.
9	72.0	0	23.57	No	No	Grade 2: Anemia.
10	58.0	0	88.86	No	No	Grade 2: Anemia;<br>Grade 1: Groin Pain.
11	75.0	0	55.71	Yes	Yes	Grade 3: Asthenia;<br>Grade 2: Leukopenia, Neutropenia, Skin infection;<br>Grade 1: Oedema peripheral.
12	82.0	0	17.29	Yes	Yes	Grade 3: Anaemia;<br>Grade 2: Asthenia, Back pain, Mucosal inflammation, Platelet count decreased;<br>Grade 1: Decreased appetite, Dyspnoea, Leukopenia, Musculoskeletal chest pain, Nausea, Neutropenia.
13	55.0	0	36.00	No	No	Grade 2: Pain;<br>Grade 1: Anaemia, Asthenia, Chest discomfort, Constipation, Mucosal inflammation, Nausea.
14	73.0	1	68.29	Yes	No	Grade 4: Bacteraemia;<br>Grade 2: Asthenia, Nausea, Nephropathy toxic, Vomiting, Decreased appetite;<br>Grade 1: Nail dystrophy, Neck pain.
